# Multifactor and multidimensional data quality analysis of judge scoring in diving competition

**DOI:** 10.3389/fpsyg.2024.1338405

**Published:** 2024-05-01

**Authors:** Weijun Cai, Rong Xiang

**Affiliations:** College of Quality and Standardization, China Jiliang University, Hangzhou, China

**Keywords:** diving competition, judge scoring, data quality, correlation, Kendall coefficient

## Abstract

**Introduction:**

In sports competitions, judge scoring data serve as an objective measure of an athlete’s performance level. However, research has indicated the unreliability of objective measurements. Controversy often arises regarding the quality of judge scoring data, undermining fairness and justice in sports competitions.

**Method:**

This paper proposes a method utilizing the Kendall covariance coefficient and the Kendall correlation coefficient for the thorough evaluation of judging data quality in diving events. The analysis is structured around four key elements: overall competition, individual divers, specific rounds, and distinct diving techniques. Each element is analyzed across three dimensions: the collective data quality from the judging panel, interjudge data quality comparisons, and the alignment of individual judges’ scores with the final tallied scores.

**Results:**

Two case studies serve to illustrate the application of this method. The Kendall covariance coefficient is employed to assess the data quality from the judges as a unified entity, whereas the Kendall correlation coefficient is utilized to evaluate the data quality from individual judges. Results show that the data quality of the judge group’s scoring is high, while the data quality of the judge group’s scoring for the 6th diver, the 5th round, Dive No. 5152B, Judge 5 and 6 in the Competition 1, and the 1st diver, the 3rd round, Dive No. 6245D, Judge 4 in the Competition 2 is inconsistent with the others.

**Discussion:**

This approach uncovers disparities in data quality attributed to the judges’ panel across each diver, each round, and the various diving maneuvers. However, the Kendall correlation coefficient may not be suitable for evaluating data quality when both the data differences and the sample size are small.

## Graphical abstract

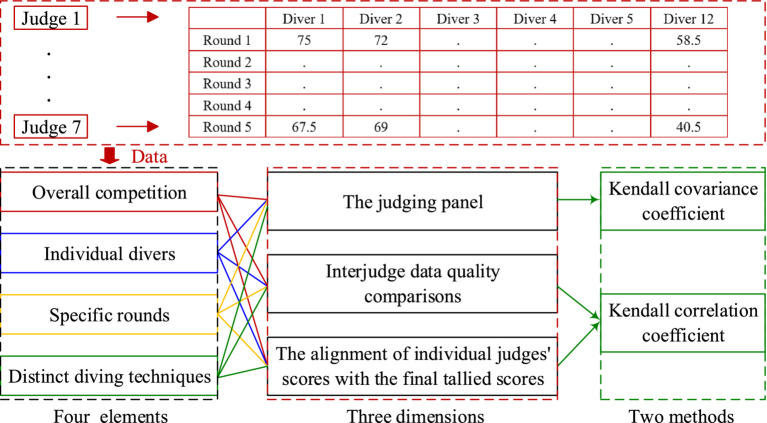

## 1 Introduction

In sports competitions, judge scoring data serve as an objective measure of an athlete’s performance level. However, research has indicated the unreliability of objective measurements (Bodington, 2017; Martire and Montgomery-Farrer, 2020; Berg et al., 2022). Controversy often arises regarding the quality of judge scoring data, undermining fairness and justice in sports competitions (Looney, 2004).

To assess the quality of judge scoring data, it is crucial to categorize it into variable and attribute data. Attribute data can be further subdivided into ordinal and categorical data. Various data quality analysis methods are employed for judging scoring assessment, including the intraclass correlation coefficient (ICC), ANOVA (Leandro et al., 2017), Spearman’s rank correlation coefficient, and Kendall covariance coefficient (Ponciano et al., 2018).

The ICC stands as a reliability indicator to gage interobserver and retest reliability. It quantifies the ratio of individual variability to total variability, with values ranging between 0 and 1. A score of 0 implies no credibility, while 1 indicates complete credibility (Cicchetti, 1994). ICC is suitable for quantitative data analysis. For instance, Pajek et al. (2013) employed the ICC to assess the reliability of scoring in the 2011 Berlin Gymnastics European Championships. The results revealed poor reliability in vault and field scores, with increased deviation observed in judge scoring during the finals of all-around and apparatus competitions. Similarly, Premelč et al. (2019) utilized the ICC to analyze scoring data from 12 pairs of adult dancers in an international competition, revealing relatively low scoring consistency among judges. Sato and Hopper (2021) also employed the ICC to assess judges’ evaluation reliability.

ANOVA, on the other hand, is employed to determine whether categorical independent variables significantly impact numerical dependent variables by testing the equality of means across populations (Jia et al., 2018). It is well suited for analyzing the relationship between categorical and quantitative data. For example, Gao (1987) conducted a two-way ANOVA (considering singers and judges) to evaluate judge scoring data quality in young singer competitions. The results indicated no significant differences in judge scoring data. Similarly, Dai and Dai (2016) utilized a two-way ANOVA with interaction (considering athletes, prescribed actions, and their interaction) to assess the performance of divers, revealing reliable judge scoring results.

The Spearman rank correlation coefficient is used to explore the correlation between two variables based on the rank differences between paired data points (Pearson and Snow, 1962). It is particularly suitable for rank variable data exhibiting linear relationships between columns. Wang et al. (1997) applied the Spearman rank correlation coefficient to classify judges’ scoring quality in national sports competitions, finding a relatively high overall level of judging in the competition.

The Kendall covariance coefficient serves as a valuable tool for assessing the correlation degree of multiple rank variables and is particularly suited for analyzing correlated rank data involving multiple columns. In a study conducted by He (2005), the Kendall Concordance Coefficient was employed to analyze the overall correlation among scores given by a panel of judges in the Men’s First Division Free Exercise Final of the National Junior Gymnastics Championships. The results indicated that the overall scoring quality of the judge group in the competition met the needed standards. Similarly, Sato (2022) utilized the Kendall covariance coefficient to evaluate the reliability of scores in annual national dance competitions held in Japan from 2014 to 2019. The findings revealed that technical scoring data exhibited the strongest correlation, while impression performance scoring data exhibited the weakest correlation.

In summary, the ICC and ANOVA are primarily utilized for the quality analysis of quantitative data. Since sports judges’ scoring data often involve countable data, the Spearman rank correlation coefficient is employed to analyze the correlation between two variables, while the Kendall covariance coefficient is utilized for analyzing the correlation between multiple variables. Currently, the application of Kendall’s coefficient of covariance in the quality analysis of judge scoring data mostly focuses on overall data quality analysis including all judges. However, it lacks a multifactor analysis considering aspects such as athletes, competition rounds, and specific movements. Additionally, there is no comprehensive analysis of data quality from various angles, including interjudge correlations and the relationship between each judge’s scores and the final score. Consequently, a comprehensive understanding of how to enhance the quality of judge scoring data is currently lacking.

The purpose of this study is to achieve a thorough multifactor and multiangle data quality analysis of judge scoring data in a single competition based on the Kendall coefficient across four dimensions: the entire diving competition, each individual athlete, each competition round, and each type of diving movement, and three key perspectives: assessing the quality of scoring data within the judge group, evaluating the quality of scoring data between two judges, and analyzing the quality of data between the scores assigned by each judge and the final overall score.

## 2 Data and methods

### 2.1 Data source and structure

The judge scoring data utilized in this study were sourced from the official website of FINA (World Aquatics, 2023a, World Aquatics, 2024), with a specific focus on the FINA Diving World Cup 2022-Women 3 m Springboard event, named the Competition 1, and the FINA Diving World Cup 2024-Men 10 m Platform event, named the Competition 2. The judge scoring data of this event is selected at random for analysis and it is a representative sample. The reason is that the scoring rules of diving competitions are similar, that is, the data acquisition methods are similar. The number of athletes, the number of judges, the number of rounds, etc. are representative, that is, the data structure is similar.

A comprehensive assessment of a diving routine includes five distinct elements: the starting position, approach, takeoff, position in height, and entry. Judges assign scores to these elements within a range of 0–10 points, taking into consideration both the technical execution and graceful performance of the divers. These scores are assigned in increments of 0.5 and are classified into seven scoring categories, namely: perfect (10 points), very good (8.5–9.5 points), good (7.0–8.0 points), average (5.0–6.5 points), poor (2.5–4.5 points), very poor (0.5–2.0 points), and failed (0 points) (Guo, 2021). The competition involves the participation of seven judges and 12 divers. Each diver undergoes five rounds of diving competitions, and the data structure for these rounds is presented in Table 1.

**Table 1:** Structure of judge scoring data for diving competition.

Judge scoring data for a diving competition						
Diver	Diving rounds	Judge 1	Judge 2	…	Judge 7	Dive Pts
Diver 1	1	8.5	8.5		8.5	75
	2	8.5	8		8.5	75
	…					
	5	8	7.5		7.5	67.5
Diver 2	1	8	8		8	72
	2	8.5	8.5		9	76.5
	…					
	5	8	7.5		8	69
…						
Diver 12	1	6.5	6.5		6.5	58.5
	2	4	3.5		4.5	37.2
	…					
	5	4.5	4.5		4.5	40.5

The scoring methodology for diving is as follows: it involves eliminating the two highest and two lowest scores among the evaluations provided by the panel of seven judges. The sum of the remaining three valid scores is then multiplied by the difficulty coefficient associated with the specific diving movement to derive the ultimate score (Guo, 2021).

### 2.2 Data analysis methods

#### 2.2.1 Data analysis dimensions

This study provides a comprehensive and systematic analysis of judge scoring data quality across four key factors: the entirety of the diving competition, individual divers, specific competition rounds, and various types of diving movements. Within each of these factors, the assessment investigates three distinct dimensions: the quality of scoring data within the judge group, the consistency of scoring data among judges, and the alignment of scoring data with the final overall score for each judge.

#### 2.2.2 Data analysis methods

In the context of diving competitions, each judge incurs a penalty of 0.5 points (Guo, 2021), rendering the judge’s scores as ordered data. This study employs the Kendall covariance coefficient to assess the overall data quality of judge group scoring. Additionally, the Kendall correlation coefficient is utilized to evaluate the quality of data between pairs of judges, as well as the alignment between each judge’s scores and the final overall score.

The Kendall covariance coefficient is particularly well suited for calculating correlations among multiple variables (Dong, 2006), as depicted in Eq. (1):


(1) $$W=\frac{12\sum_{j=1}^{k}R_{+j}^{{*}^{2}}-3b^{2}k{\left(k+1\right)}^{2}}{b^{2}k\left(k^{2}-1\right)-b\sum_{i=1}^{b}\sum_{h=1}^{g_{i}}\left(t_{h}^{3}-t_{h}\right)}$$

where $R_{+j}^{*}$ is the rank determined by the average rank method; *b* is the number of judges, which is 7; *k* is the number of divers, which is 12; $t_{h}$ is the knot length of the *h*th group; and $g_{i}$ is the number of knots in the data column obtained from the *i*th judge scoring *k* divers. Rank refers to the order of each data in its entire data column after arranging it in order of size. If the samples are equal, putting the same samples together is called a knot. The number of samples in the knot is called the knot length. When the result is greater than 1, the rank of the sample is the average of the same sample ranks, and this method is called the average rank method.

The Kendall correlation coefficient is applicable to the calculation of the correlation between two variables (Dong, 2006), as shown in Eq. (2):


(2) $$K=\frac{2\sum_{i}^{n}\sum_{j}^{n}\mathrm{sgn}\left(X_{i}-X_{j}\right)}{\sqrt{n\left(n-1\right)-\sum t_{x}\left(t_{x}-1\right)}\;\sqrt{n\left(n-1\right)-\sum t_{y}\left(t_{y}-1\right)}},i<j$$

where $t_{x}$ is the length of each knot of the *X* variable; $t_{y}$ is the length of each knot of the *Y* variable; and the Sign function is shown in Eq. (3):


(3) $$\mathrm{sgn}\left(t\right)=\{\begin{array}{c} 1,t>0\\ 0,t=0\\ -1,t<0 \end{array}$$

The Kendall covariance coefficient values fall within the range of [0, 1]. The closer the coefficient is to 0, the lower the data quality, whereas a value closer to 1 indicates higher data quality. Similarly, the Kendall correlation coefficient varies between [−1, 1], where a value nearing −1 implies lower data quality, while a value approaching 1 signifies higher data quality.

## 3 Results and discussion

### 3.1 Data quality analysis of judge scoring for the entirety of the diving competition

In this analysis, each judge’s 60 scores for the 12 divers across five rounds of diving were treated as a data column representing one variable. There were a total of seven judges, each corresponding to one of these variables. Additionally, the final scores of the 12 divers across the five rounds of diving were utilized as the data column for the eighth variable. This comprehensive approach allowed for an assessment of data quality in judge scoring across the 12 divers and five rounds of diving.

#### 3.1.1 Quality of scoring data within the judge group for the entirety of the diving competition

The Kendall covariance coefficient was calculated among the seven data columns representing the scores provided by the seven judges. This coefficient serves as an indicator for evaluating the overall data quality within the judge group’s scores.

The computed Kendall covariance coefficients, denoted as W^*^, are 0.93 and 0.96, and the *p* values obtained from the significance test are 6.68 × 10^−11^ and 2.54 × 10^−7^ for the Competition 1 and 2. These results suggest a strong correlation among the data columns of the seven judges, indicating that the overall data quality of the judge group’s scores is excellent.

#### 3.1.2 Consistency of scoring data among judges for the entirety of the diving competition

**Figure 1:**
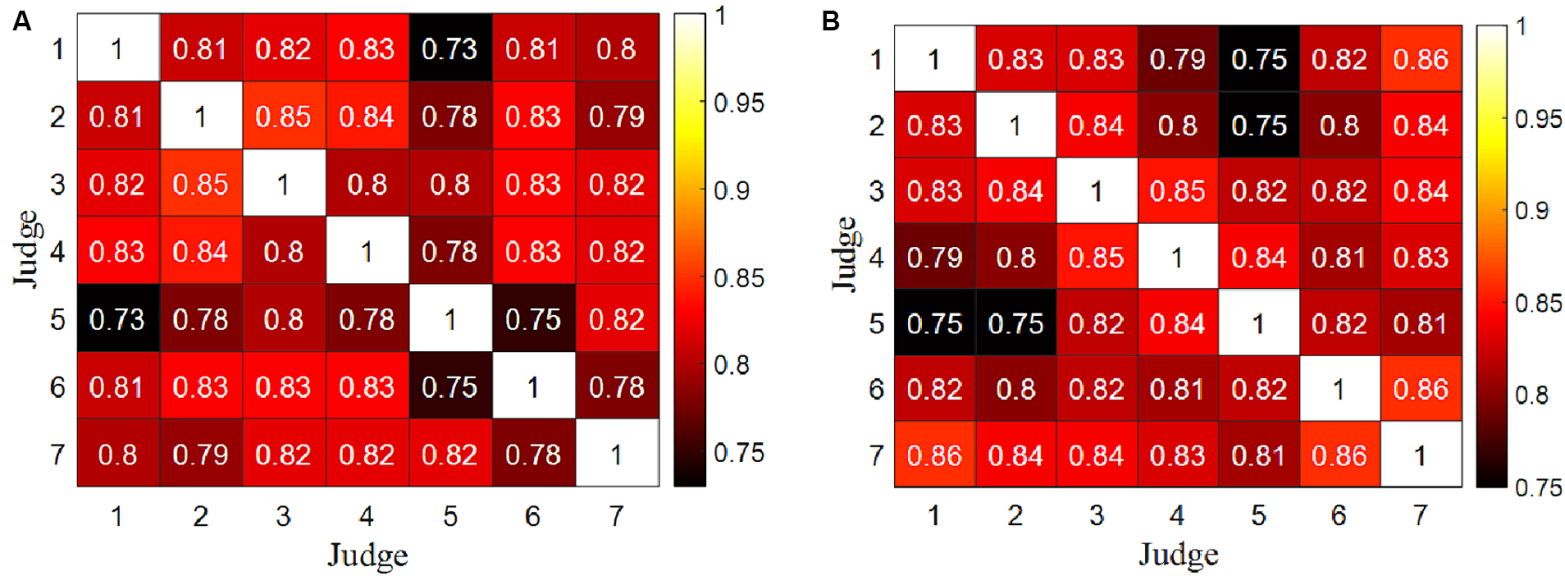
Kendall correlation coefficient matrix between two data columns of any two judges for all divers. **(A)** Competition 1. **(B)** Competition 2.

Kendall correlation coefficients were calculated between the data columns of any two judges’ scores, serving as an indicator to assess the data quality between the scores provided by any pair of judges for all divers. The results of two competitions are presented in Figure 1, which reveals that all Kendall correlation coefficients exceed 0.70, and their associated *p* values are all below 0.05. These findings suggest that the data quality between the scores provided by any two judges is relatively high.

#### 3.1.3 Alignment of scoring data with the final overall score for each judge for the entirety of the diving competition

**Figure 2:**
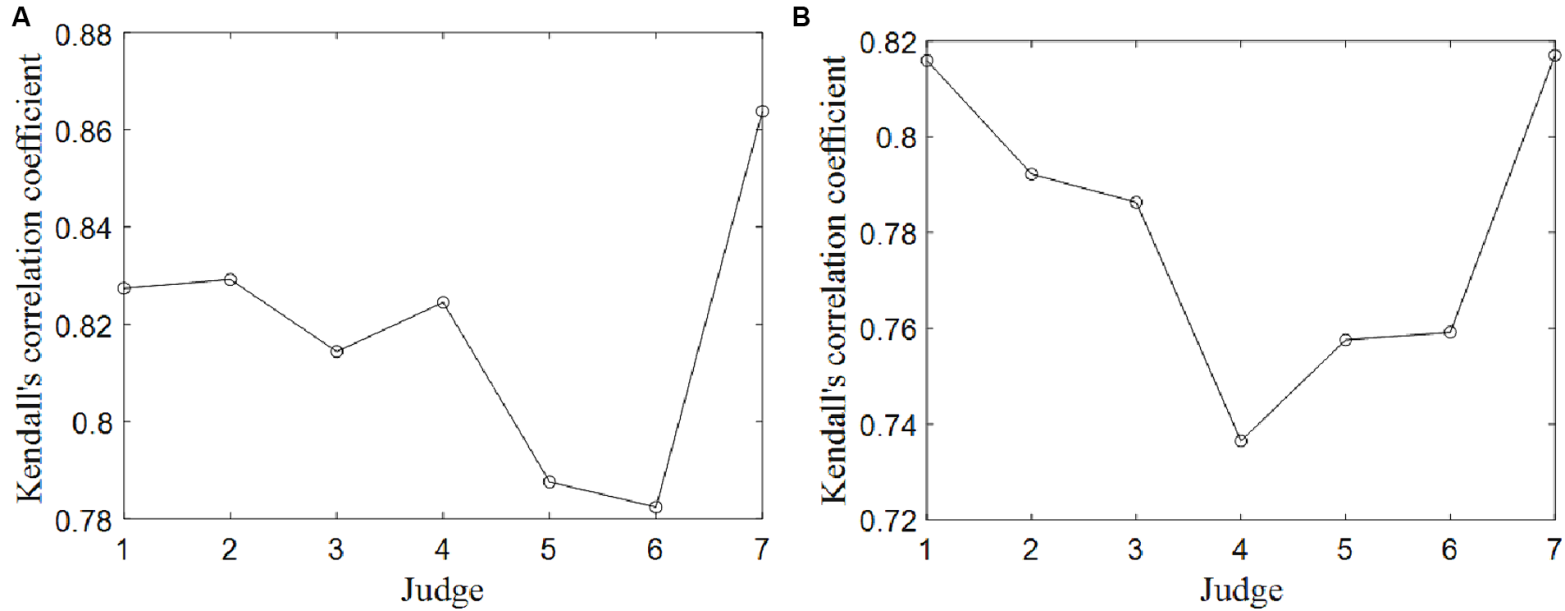
Kendall correlation coefficients between any judge’s scores and final scores for all divers. **(A)** Competition 1. **(B)** Competition 2.

Kendall correlation coefficients were computed between each judge’s scores and the final scores, serving as an indicator to assess the data quality between each judge’s score and the final scores for all divers. The results of two competitions are displayed in Figure 2.

The majority of the Kendall correlation coefficients exceed 0.75, with associated *p* values all falling below 0.05. These findings indicate that the data quality of scores provided by the seven judges is notably high. However, in comparison to the other judges, Judge 5 and 6 in the Competition 1 and Judge 4 in the Competition 2 exhibit the lowest scoring data consistency, with Kendall correlation coefficients of 0.79 and 0.78 in the Competition 1, and 0.74 in the Competition 2, respectively. Conversely, Judge 7 in the Competition 1 and Judge 1 in the Competition 2 demonstrate the highest scoring data consistency, with Kendall correlation coefficients of 0.86 and 0.82.

### 3.2 Data quality analysis of judge scoring for individual divers

In this analysis, each judge’s five scores for each diver across their five rounds of diving were treated as a data column representing one variable. There were a total of seven judges, each corresponding to one of these variables. Additionally, the final scores for each diver’s five rounds of diving were utilized as the data column for the eighth variable. This approach allowed for the assessment of data quality in judge scoring for each of the 12 divers individually.

#### 3.2.1 Quality of scoring data within the judge group for individual divers

**Figure 3:**
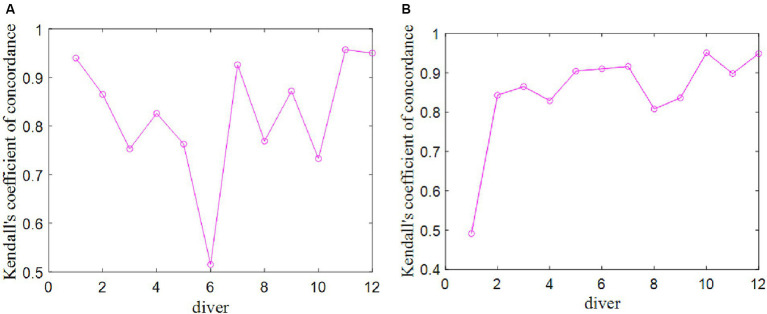
Kendall concordance coefficients of the judge group’s scores for each diver. **(A)** Competition 1. **(B)** Competition 2.

The Kendall covariance coefficients among the seven data columns representing the scores provided by seven judges were calculated. These coefficients serve as the evaluation indicators for assessing the data quality within the group of judges’ scores for each diver. It is important to note that there are a total of 12 Kendall covariance coefficients, corresponding to the 12 divers under consideration. The results of these computations are presented in Figure 3.

It is evident that all *p* values are below the threshold of 0.05, signifying a robust correlation among the scores provided by the seven judges for each individual diver. Consequently, the data quality within the group of judges’ scores is deemed to be high. Notably, for the sixth diver in the Competition 1 and the first diver in the Competition 2, the Kendall coefficient associated with the judge group’s scores is the smallest, indicating that, in the case of the sixth diver in the Competition 1 and the first diver in the Competition 2, the data quality of the judge group’s scores is comparatively lower.

When considering the highest and lowest ranked divers, it is observed that the data quality of the judge group’s scores is relatively high, with Kendall correlation coefficients exceeding 0.85. This can be attributed to the fact that judges consistently applied deductions of 0.5–2 points for nonstandard actions, adhering to the defined standards for scoring actions as outlined in the scoring rules (World Aquatics, 2023b). For these top-and bottom-ranked divers, the judges were able to clearly assess the quality of their movements in accordance with the scoring standards.

Conversely, in regard to the divers with middle rankings, there appears to be more ambiguity in the judges’ assessments, leading to inconsistent deductions. This inconsistency in judgment contributes to a higher level of uncertainty in the scoring data quality for divers occupying intermediate positions in the rankings.

#### 3.2.2 Consistency of scoring data among judges for individual divers

**Figure 4:**
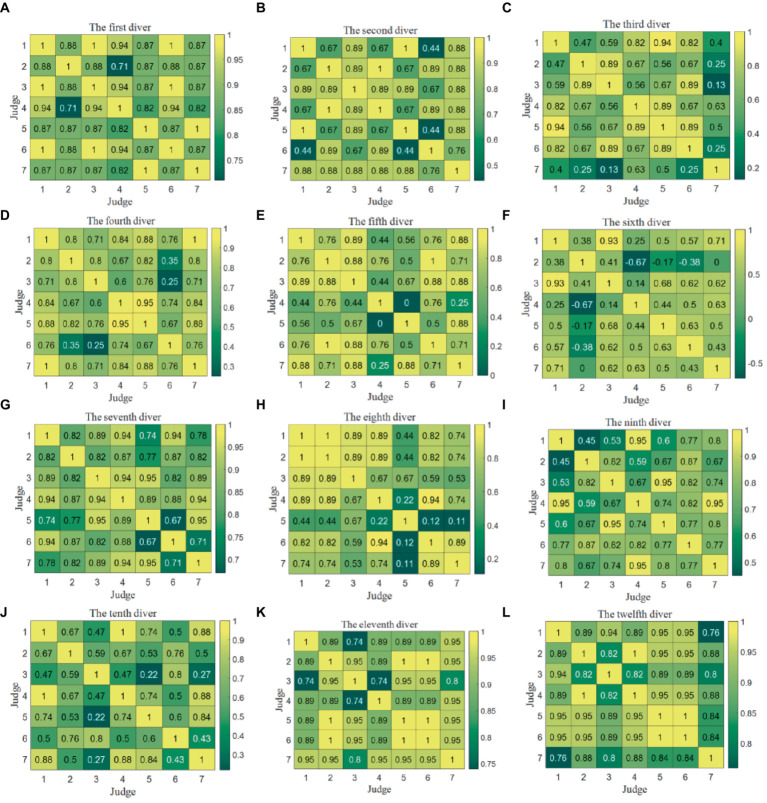
Kendall correlation coefficient matrices between any two judges’ scores for each diver in the Competition 1. **(A)** The first diver. **(B)** The second diver. **(C)** The third diver. **(D)** The fourth diver. **(E)** The fifth diver. **(F)** The sixth diver. **(G)** The seventh diver. **(H)** The eighth diver. **(I)** The ninth diver. **(J)** The tenth diver. **(K)** The eleventh diver. **(L)** The twelfth diver.

The Kendall correlation coefficients were computed between pairs of data columns representing the scores provided by any two judges. These coefficients serve as an essential evaluation indicator for assessing the data quality between the scores of any two judges for each individual diver. It is important to note that there exist 12 Kendall correlation coefficient matrices, each corresponding to one of the 12 divers, as depicted in Figure 4. Due to the similar analysis for two competitions and the space limitation, this section only analyzes the judge scoring quality in the Competition 1.

It is evident that in the case of the sixth diver, the Kendall correlation coefficient between Judge 2 and Judges 4–6 falls below 0, while the correlation coefficient between Judge 2 and Judge 7 stands at 0. This discrepancy arises from the fact that Judge 2 assigned identical scores to 4 instances, leading to a divergent ranking of the five scores in comparison to the assessments made by the other judges. Consequently, this results in Kendall correlation coefficients among the judges that are either less than or equal to 0.

In truth, the disparities in the data were not substantial, with variations of 1.5 points or less. This observation underscores that the Kendall correlation coefficient may not be the most appropriate metric for evaluating data quality when both the data differences and the sample size are minimal. Additionally, it is worth noting that numerous Kendall correlation coefficients equate to 1, signifying that different judges share the same ranking while assigning distinct values to a particular diver. This emphasizes that a strong correlation does not necessarily imply good consistency.

#### 3.2.3 Alignment of scoring data with the final overall score for each judge for individual divers

**Figure 5:**
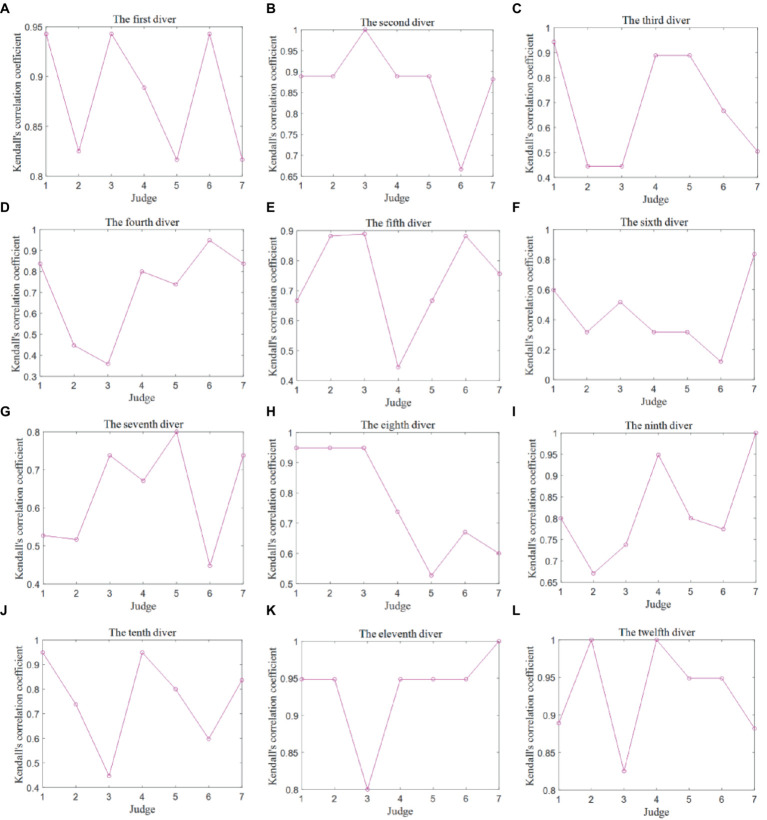
Kendall correlation coefficients between any judge’s scores and final scores for each diver in the Competition 1. **(A)** The first diver. **(B)** The second diver. **(C)** The third diver. **(D)** The fourth diver. **(E)** The fifth diver. **(F)** The sixth diver. **(G)** The seventh diver. **(H)** The eighth diver. **(I)** The ninth diver. **(J)** The tenth diver. **(K)** The eleventh diver. **(L)** The twelfth diver.

The Kendall correlation coefficients were computed between each judge’s score data column and the data column of the final scores. These coefficients serve as a crucial metric for evaluating the data quality between each judge’s scores and the final scores for each of the 12 divers. Due to the similar analysis for two competitions, only the judge scoring quality in the Competition 1 was analyzed. It is important to note that there exist 12 Kendall correlation coefficient charts, each corresponding to one of the 12 divers, as depicted in Figure 5.

In some instances, for certain divers, the Kendall correlation coefficients between some judges’ scores and the final scores are equal to 1, even when there are differences between the judge’s scores and the final scores. This phenomenon indicates that for these divers, there is a strong correlation between the judge’s scores and the final scores, but it does not necessarily imply a high level of consistency. This further underscores the notion that the Kendall correlation coefficient may not be the most suitable metric for analyzing data with small numerical differences and limited sample sizes.

**Figure 6:**
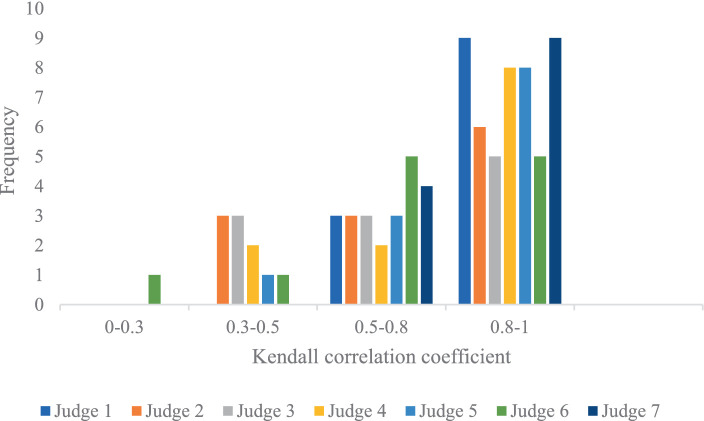
Frequency of *K* values between any judge’s scores and final scores for each diver.

To provide a more comprehensive analysis of the data quality in the context of different judges’ scoring, the correlation coefficient *K* was categorized into four groups: *K* ≥ 0.8 signifies a high correlation; 0.5 ≤ *K* < 0.8 indicates a moderate correlation; 0.3 ≤ *K* < 0.5 suggests a low correlation; and *K* < 0.3 indicates an extremely weak correlation, which can be considered uncorrelated (Dong, 2006). The frequency of *K* values for the seven judges in relation to each diver was computed and is presented in Figure 6.

The analysis reveals that, for each diver, the Kendall correlation coefficient predominantly falls within the range of 0.8–1, indicating that the quality of the judges’ scores is relatively high. Additionally, Judge 1 and Judge 7 exhibit the highest frequency of *K* values with high correlation, while Judge 3 and Judge 6 display the lowest frequency in this category.

### 3.3 Data quality analysis of judge scoring for specific competition rounds

In each round of diving, a set of 12 scores from each of the seven judges for the 12 divers was treated as a data column representing a single variable. These seven judges were considered as seven distinct variables, and the final scores for the 12 divers in each round of diving were utilized as the data column for the eighth variable. The assessment of data quality for the judges’ scoring was conducted separately for each of the five rounds.

#### 3.3.1 Quality of scoring data within the judge group for specific competition rounds

**Figure 7:**
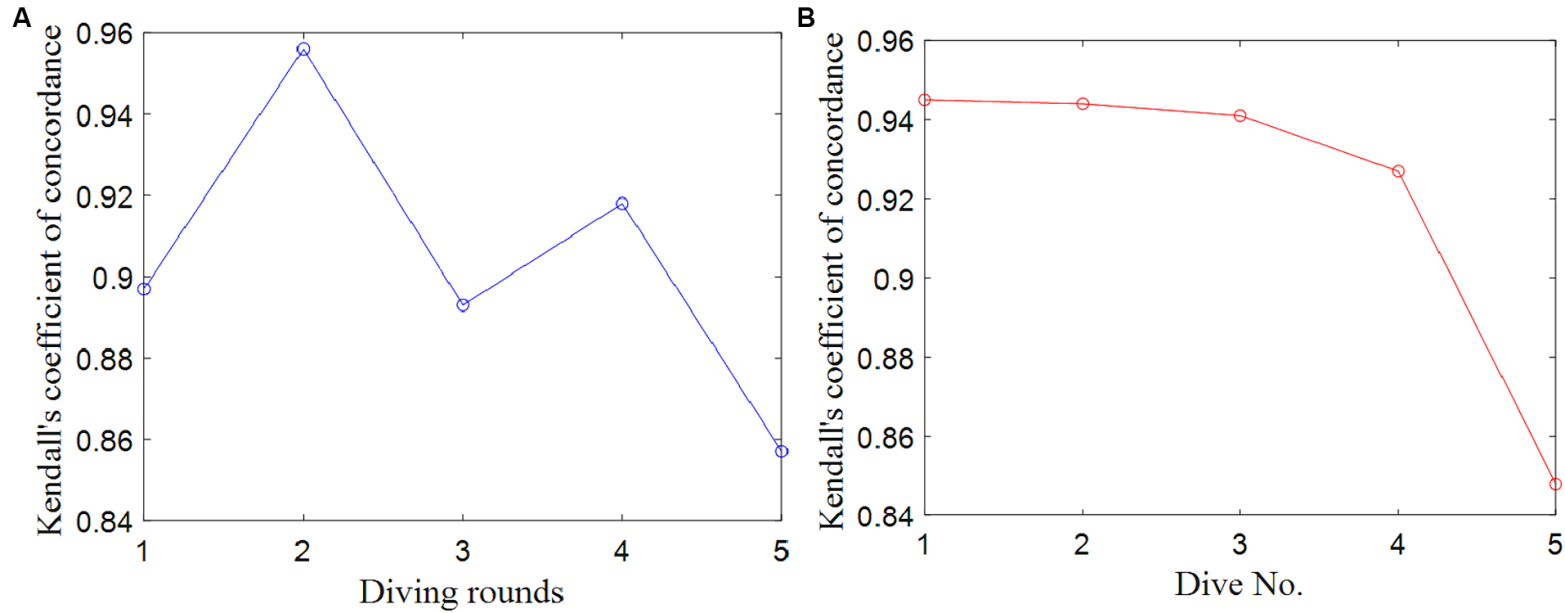
Kendall concordance coefficient of the judge group’s scores for each round and dive No. **(A)** Each round. **(B)** Dive No.

The Kendall covariance coefficients were computed among the seven data columns representing the scores given by the seven judges for each round of diving. These coefficients were employed as evaluation indicators for assessing the data quality within the group of judges’ scores for each round. In total, there are five Kendall covariance coefficients, each corresponding to one of the five rounds, as depicted in Figures 7A,B.

It is worth noting that all the Kendall covariance coefficients (W^*^) are consistently greater than 0.80, and the associated *p* values are uniformly less than 0.05 in two competitions. These findings indicate that the data quality of the judge group’s scores for each round of diving is relatively high. However, it is noteworthy that the data quality for the fifth round in the Competition 1 and the third round in the Competition 2 appear to be comparatively lower, as indicated by a Kendall covariance coefficient of 0.86 and 0.81.

#### 3.3.2 Consistency of scoring data among judges for specific competition rounds

**Figure 8:**
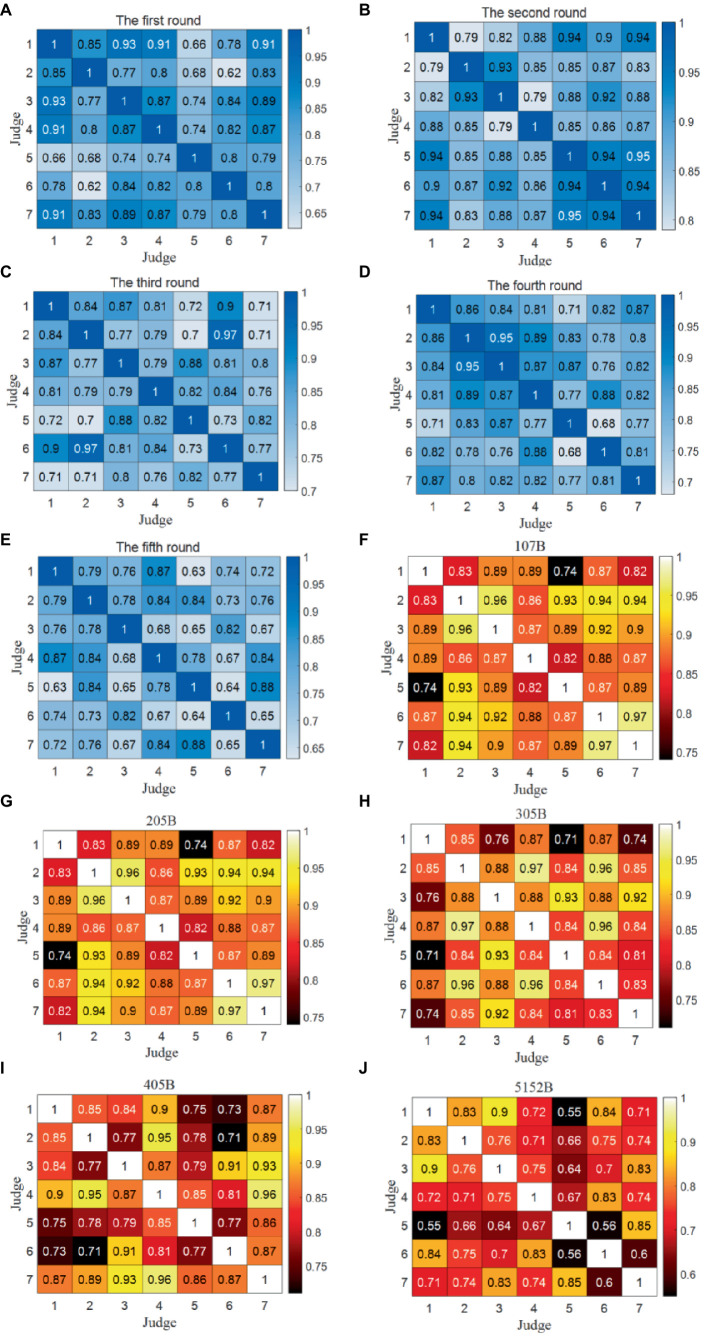
Kendall correlation coefficient matrices between any two judges for each round and movement in the Competition 1. **(A)** The first round. **(B)** The second round. **(C)** The third round. **(D)** The fourth round. **(E)** The fifth round. **(F)** 107B. **(G)** 205B. **(H)** 305B. **(I)** 405B. **(J)** 512B.

The Kendall correlation coefficients were calculated between the data columns representing the scores provided by any two judges, serving as a key metric to evaluate the data quality between any pair of judges’ scores for each round. There exist five Kendall correlation coefficient matrices, each corresponding to one of the five rounds, as displayed in Figures 8A–E. Here only shows the result of the Competition 1 because of the similar analysis for two competitions.

It is noteworthy that all associated *p* values are consistently less than 0.05, indicating a positive correlation between the scores assigned by any two judges. However, certain rounds reveal notable variations in the Kendall correlation coefficients:

In the first round, the Kendall correlation coefficients between Judge 5 and Judges 1 and 2 and between Judge 6 and Judge 2 are all less than 0.70. This suggests significant scoring differences among these judges in the first round.

In the fourth round, the Kendall correlation coefficient between Judge 5 and Judge 6 falls below 0.70, indicating a significant scoring disparity between these two judges during the fourth round.

In the fifth round, the Kendall correlation coefficients between Judge 5 and Judges 1, 3, and 6, between Judge 6 and Judges 4 and 7, and between Judge 3 and Judges 4 and 7 are all less than 0.70. This implies significant scoring differences among these judges in the fifth round.

In summary, the analysis reveals that Judge 5 and Judge 6 exhibit the lowest scoring data consistency, as evidenced by the consistent significant differences in their scoring patterns across multiple rounds.

#### 3.3.3 Alignment of scoring data with the final overall score for each judge for specific competition rounds

**Figure 9:**
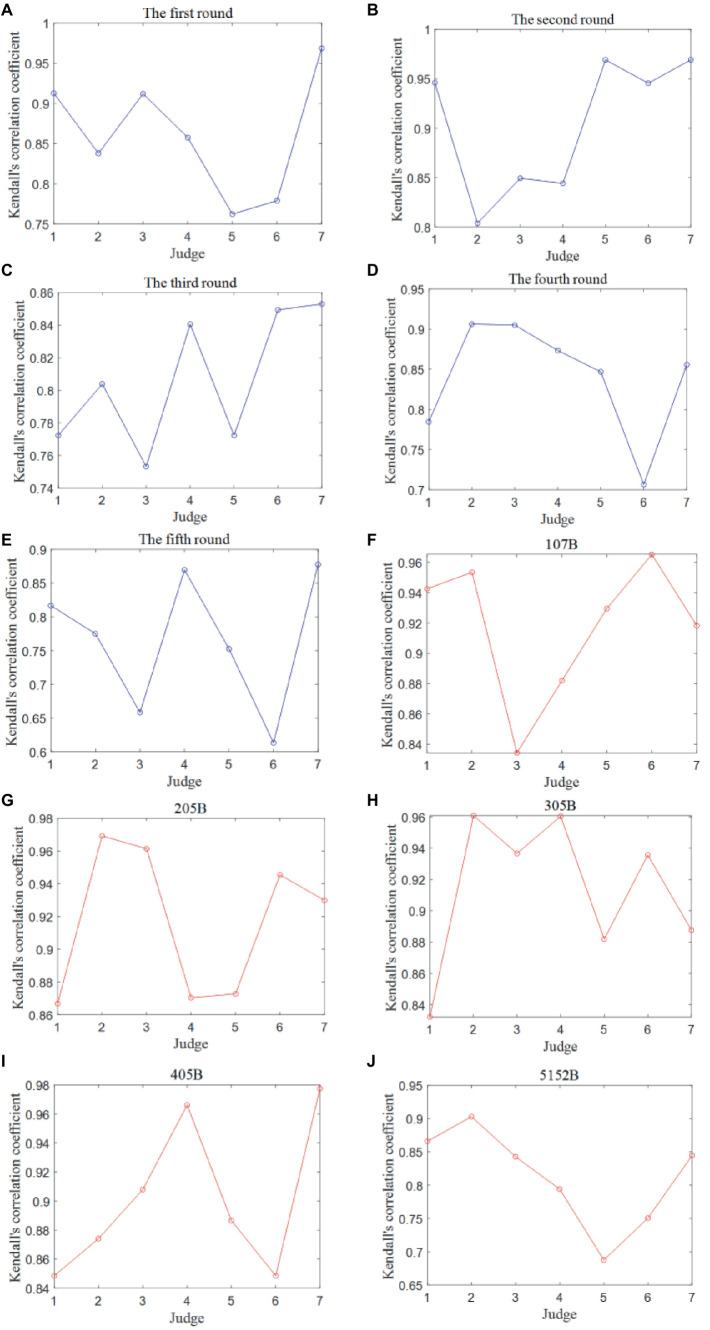
Kendall correlation coefficient between any judge’s scores and final scores for each round and movement in the Competition 1. **(A)** The first round. **(B)** The second round. **(C)** The third round. **(D)** The fourth round. **(E)** The fifth round. **(F)** 107B. **(G)** 205B. **(H)** 305B. **(I)** 405B. **(J)** 512B.

The Kendall correlation coefficients between the data column of any judge’s scores and the data column of final scores were computed and were regarded as an evaluation indicator of the data quality between any judge’s score and final scores for each round. There are five Kendall correlation coefficient charts for five divers, as shown in Figures 9A–E. Only the results of the Competition 1 were analyzed in this section because of the similar analysis for two competitions. The Kendall correlation coefficients are all greater than 0.7, and the *p* values are all less than 0.05, indicating that the quality of scoring data for each judge in each round is relatively high.

Compared to other judges, Judge 5 has the lowest Kendall correlation coefficient in the first round, while Judge 6 has the lowest Kendall correlation coefficient in the fourth and fifth rounds. This proves that the consistency of scoring data for Judge 5 and Judge 6 is poorer than the others once again. In addition, Judge 2 has the worst scoring data consistency in the second round but had the best scoring data quality in the fourth round, reflecting the unstable consistency of Judge 2’s scoring data.

### 3.4 Data quality analysis of judge scoring for various types of diving movements

In the Competition 1, 12 divers selected a total of eight types of diving movements in five rounds, namely, dive No. 107B, 107C, 205B, 305B, 405B, 405C, 5152B, and 5152C. Due to the limited number of choices selected by divers for dive No. 107C, 405C, and 5154B, the remaining five diving movements were selected for judge scoring data quality analysis. In the Competition 2, six types of diving movements, namely, 109C, 207C, 307C, 407C, 5255B, and 6245D, were selected by 12 divers.

The scores from each judge for all divers who chose each type of diving movement were regarded as a data column of one variable. Seven judges were regarded as seven variables, and the final scores of 12 divers in each round of diving were used as the data column of the eighth variable. The quality of the judge scoring data for each type of diving movements was analyzed.

#### 3.4.1 Quality of scoring data within the judge group for various types of diving movements

The Kendall covariance coefficients among the seven data columns of seven judges’ scores were computed and were regarded as the evaluation indicators of the data quality among the judge group’s scores for each diving movement. There are a total of 5 and 6 Kendall covariance coefficients for 5 and 6 types of diving movements in the Competition 1 and 2. The results are shown in Figures 7C,D and Tables 2, 3.

**Table 2:** Kendall concordance coefficients of five diving movements in the Competition 1.

Dive no.	Diving movement	Frequency	Degree of difficulty	*W* ^*^
107B	Pike forward triple somersaults	10	3.1	0.95
205B	Pike back 2 1/2 somersaults	12	3.0	0.94
305B	Pike 2 1/2 gainer	12	3.0	0.94
405B	Pike inward 2 1/2 somersaults	10	3.0	0.93
5152B	Pike inward 2 1/2 somersaults and full twist	11	3.0	0.85

**Table 3:** Kendall concordance coefficients of five diving movements in the Competition 2.

Dive no.	Diving movement	Frequency	Degree of difficulty	*W* ^*^
109C	Crouched jump forward 4 1/2	11	3.7	0.96
207C	Crouched jump back 4 1/2	7	3.3	0.88
307C	Crouched jump reverse 4 1/2	12	3.4	0.97
407C	Crouched jump inward 4 1/2	12	3.2	0.90
5255B	Pike back 2 1/2 and twist 2 1/2	6	3.6	0.94
6245D	Armstand dive 2 1/2 and twist 2 1/2	8	3.6	0.67

In the two competitions, the Kendall covariance coefficients were above 0.85 and 0.65, and the *p* values were all less than 0.05, indicating that for the all of diving movements, the data quality of the judge group’s scores was relatively high. The W^*^ of 5152B is the smallest in the Competition 1 and the W^*^ of 6245D is the smallest in the Competition 2, which is 0.85 and 0.67, indicating that the quality of the judge group scoring data for Dive No. 5152B is the worst in the Competition 1 and that for Dive No. 6245D is the worst in the Competition 2. Since most divers chose 5152B in the fifth round, the quality of the judge group scoring data of the fifth round was the worst, which is consistent with the conclusion of the judge group scoring data quality for each round.

#### 3.4.2 Consistency of scoring data among judges for various types of diving movements

The Kendall correlation coefficients were computed between the data columns representing the scores provided by any two judges, serving as a vital metric to evaluate the data quality between any pair of judges’ scores for each of the five diving movements. There exist five Kendall correlation coefficient matrices of the Competition 1, each corresponding to one of the five diving movements, as presented in Figures 8F–J.

It is noteworthy that all associated *p* values are consistently less than 0.05, indicating a positive correlation between the scores assigned by any two judges for each diving movement. However, specific findings within the analysis stand out:

For Dive No. 5152B, the correlation between Judge 5 and Judges 1–4, and 6 is relatively poor. This suggests that these judges exhibit some differences in their evaluation criteria for Dive No. 5152B.

In the case of Judge 6, the Kendall correlation coefficients with Judge 7 are all less than 0.70. This indicates that Judge 6 and Judge 7 have notable differences in their scoring assessments, further emphasizing discrepancies in scoring criteria.

Once again, these observations reinforce the notion that Judge 5 and Judge 6 consistently exhibit the lowest scoring data consistency, with notable disparities in their scoring assessments across various diving movements.

#### 3.4.3 Alignment of scoring data with the final overall score for each judge for various types of diving movements

The Kendall correlation coefficients were computed between each judge’s score data column and the data column of the final scores, serving as a valuable metric to assess the data quality between each judge’s scores and the final scores for each of the five diving movements. There exist five Kendall correlation coefficient charts of the Competition 1, each corresponding to one of the five diving movements, as depicted in Figures 9F–J.

It is notable that all associated *p* values consistently fall below 0.05, indicating a positive correlation between the scores assigned by any two judges for each type of diving movement. However, specific observations within the analysis reveal variations in the data quality among judges for different diving movements:

For Dive No. 107B, Judge 3 demonstrates the lowest data consistency in terms of scores given. In the case of Dive No. 205B, Judge 1 exhibits the lowest data consistency in scoring. For Dive No. 305B, Judge 1 displays the poorest scoring data consistency. In the context of Dive No. 405B, Judges 1 and 6 both have the lowest scoring data consistency. Regarding Dive No. 5152B, Judge 5’s scoring data consistency is the least satisfactory.

The analysis highlights that Dive No. 5152B, characterized by more complex twist movements compared to the other dives, poses a challenge to judges and leads to increased subjectivity in scoring, resulting in suboptimal data quality in their assessments. This underscores the importance of refining diving competition rules, deduction rules, and scoring criteria for each movement and clarifying standards and nonstandard deduction scores to reduce the subjectivity in judges’ scoring.

Compared with the existing research which only analyzed the collective data quality from the judging panel, the method proposed in this study can be used to analyze the scoring quality from different perspectives such as overall competition, individual divers, specific rounds, and distinct diving techniques. On the other hand, this study also conducted a multidimensional analysis of the scoring quality from the judging panel, interjudge data quality comparisons, and the alignment of individual judges’ scores with the final tallied scores to analyze the scoring quality of each judge more finely, but not just analyzing the scoring quality of the judging panel.

Based on the application of the method, the sports authorities can rate and select the judge according to the judge scoring data quality, and choose the judge with high scoring data quality. If the profile of the judge in each competition is available, the judge scoring quality evaluation results of all competitions for each judge can be recorded, so that the judge with persistently low scoring quality can be demoted and the judge with persistently high scoring quality can be upgraded. The technical committee may evaluate the clarity, rationality and the consistency of the judges’ understanding of the scoring rules according to the judge scoring data quality of each diving technique, and improve the scoring rules for diving techniques with low judge scoring quality. Coaches and athletes can analyze the maturity of diving technique completion according to the judge scoring quality of each athlete. For athletes with low judge scoring quality, there are inconsistent evaluations among different judges on the quality of diving technique completion. After the analysis of diving techniques, the diving technique completion degree and the conformity degree with the scoring rules can be improved. The above application plays a positive role in improving the fairness of the competition and improving the level of the athletes.

In future research, the proposed method can be applied to other competitions, as well as to the competition with technology-assisted decision, and then the difference between the judge scoring quality in the two situations with and without technology-assisted decision will also be compared and analyzed. To solve the problem caused by the small difference in judges’ scores, which affects the ranking of athletes, thus leading to the difference in scoring quality among judges, we can regard judge’s scores as quantitative data, and use variation analysis to analyze the ratio of different judges’ score variation to the same athlete and different athletes’ score variation to realize the judge scoring data quality analysis.

## 4 Conclusion

1. Variations in data quality are observed in the judge group’s scoring for divers with different rankings. Divers at both ends of the ranking spectrum exhibit higher data quality in judge scoring compared to divers in the middle. Specifically, in this case, the data quality of the judge group’s scoring for the sixth diver in the Competition 1 and the first diver in the Competition 2 is the lowest separately.
2. Disparities in data quality are evident in the judge group’s scoring for different rounds. Notably, the data quality of the judge group’s scoring for the fifth round in the Competition 1 and the third round in the Competition 2 is the least satisfactory separately.
3. The data quality of the judge group’s scoring varies across different diving movements. In this instance, the data quality of the judge group’s scoring for Dive No. 5152B in the Competition 1 and Dive No. 6245D in the Competition 2 is the poorest separately.
4. The overall data quality of the judge group’s scoring can be quantitatively analyzed using the Kendall concordance coefficient. Additionally, the data quality of each judge’s scoring can be assessed quantitatively through the Kendall correlation coefficient between any two judges’ scores or between each judge’s scores and the final scores. In this case, the data quality of the judge group’s scoring is high, while the scoring data consistency of Judge 5 and 6 in the Competition 1 and Judge 4 in the Competition 2 is relatively subpar separately.
5. The Kendall correlation coefficient may not be suitable for evaluating data quality when both the data differences and the sample size are small.

## Data availability statement

Publicly available datasets were analyzed in this study. This data can be found at: https://www.worldaquatics.com/competitions/3012/finadiving-world-cup-2022/results?event=1ea405af-8ac1-4046-ba0f-c2b1a11615c3 and https://www.worldaquatics.com/competitions/3377/world-aquatics-diving-world-cup-2024/results?event=54bcb578-a91f-4f52-af7b-63217e281d6b.

## Author contributions

CW: Visualization, Writing – original draft. XR: Conceptualization, Methodology, Resources, Supervision, Validation, Writing – review & editing.
